# Inhibition of hyaluronan export reduces collagen degradation in interleukin-1 treated cartilage

**DOI:** 10.1186/ar2357

**Published:** 2008-01-18

**Authors:** Barthold Deiters, Peter Prehm

**Affiliations:** 1 Muenster University Hospital, Institute of Physiological Chemistry and Pathobiochemistry, Waldeyerstr. 15, D-48129 Münster, Germany

## Abstract

**Background:**

Osteoarthrosis is characterized by cartilage erosion, proteolysis of aggrecan and collagen, and disturbed rates of synthesis of aggrecan and hyaluronan by chondrocytes, with hyaluronan over-production being an early reaction. We considered that inhibition of hyaluronan export might prevent subsequent proteoglycan loss and collagen degradation.

**Methods:**

To test this hypothesis, we studied a tissue culture model using bovine cartilages explants activated with IL-1α to induce osteoarthritic reactions using the phosphodiesterase-5 inhibitors tadalafil, zaprinast and vardenafil.

**Results:**

These drugs inhibited hyaluronan export, but they did not inhibit hyaluronan synthase activity. Simultaneously, they inhibited proteoglycan loss and collagen degradation, but not their synthesis. They also reduced the release of gelatinases into the culture media and diffusion of the indicator protein horseradish peroxidase through the cartilage explants. The mechanism of action of these compounds may be through inhibition of hyaluronan exporter multidrug resistance-associated protein 5 (MRP5), because the effective drug concentrations were much higher than required for phosphodiesterase-5 inhibition and intracellular cGMP accumulation.

**Conclusion:**

Inhibition of hyaluronan over-production may be an appropriate target to attenuate IL-1-induced reactions in osteoarthritic cartilage.

## Introduction

Destruction of joint cartilage is the major outcome of arthritic diseases such as osteoarthrosis and rheumatoid arthritis. Although chondrocytes represent only 5% of the tissue, these cells are responsible for cartilage matrix synthesis, which consists of two main components: the network of type II collagen, which provides the tensile strength and stiffness; and the large aggregating proteoglycan aggrecan, which is responsible for the osmotic swelling capability and elasticity. Aggrecan decorates a backbone of hyaluronan that is partially anchored in the plasma membrane of chondrocytes at the hyaluronan synthase site and is further bound by the cell surface receptor CD44. Aggregate formation is important from a physiological point of view because it ensures the retention of aggrecan within the collagen network.

The biosyntheses of hyaluronan and proteoglycans take place via different mechanisms and occur in different compartments [1]. Proteoglycans are synthesized in the Golgi and exocytosed by vesicles. Hyaluronan is polymerized at the inner side of plasma membranes [1-4] and was originally thought to be exported by the synthase itself [5,6], but recently the ATP-binding cassette transporter multidrug resistance protein (MRP)5 was identified as a hyaluronan exporter [7,8]. Both components aggregate in the extracellular matrix [9], with up to 200 aggrecan molecules decorating one hyaluronan chain [10]. In healthy cartilage, the hyaluronan and aggrecan are synthesized and degraded at similar rates [11], whereas the turnover of collagens is much slower [12]. The proteoglycan monomer is liberated from the hyaluronan binding region by aggrecanases, matrix metalloproteases and cathepsins [13-17]. In healthy cartilage, most of hyaluronan is removed by endocytosis through the CD44 receptor [18], whereas in osteoarthritic cartilage about 90% is liberated into the environment [19]. Aggrecan leaves cartilage either as intact molecule or after proteolysis, depending on the stimulus [20].

Key events in osteoarthritic cartilage are increased hyaluronan, decreased aggrecan synthesis [19,21], and proteolytic cleavage of collagen type II and aggrecan core protein [22,23]. For a long time it was believed that proteolytic degradation of collagen and aggrecan was the primary event in cartilage breakdown. Much effort to develop protease inhibitors led to compounds that were chondroprotective *in vitro *or in animal models [24-27], but the findings of clinical trials were equivocal [28,29].

Recently, we discovered that a variety of multidrug resistance inhibitors interfered with hyaluronan export by the the multidrug resistance-associated protein MRP5 [7,8]. Some of the hyaluronan export inhibitors have already been applied to prevent hyaluronan over-production and proteoglycan loss in IL-1α activated chondrocyte cell cultures, in cartilage organ cultures and in an animal model of osteoarthrosis [30]. Because hyaluronan export by MRP5 is regulated by intracellular cGMP [8] (also an MRP5 substrate [31]), we evaluated the effects of the drugs zaprinast, vardenafil and tadalafil. These agents are structural analogues of cGMP that inhibit the cGMP-specific phosphodiesterase (PDE5) at nanomolar concentrations [32] and so they increase intracellular cGMP levels. In addition, zaprinast is also known as a MRP5 inhibitor [33]. We analyzed their effects on hyaluronan export, proteoglycan loss and collagen degradation in IL-1α activated bovine articular cartilage explants.

## Materials and methods

### Materials

Articular cartilage was obtained from the knees of 2-year-old steer provided by a local slaughterhouse. Vardenafil was from Bayer AG (Leverkusen, Germany), tadalafil was from Elli Lilly (Indianapolis, IA, USA), hyaluronan binding protein (HABP) was from Calbiochem (Schwalbach, Germany), and hyaluronan (Healon^®^) was a gift from Genzyme (Cambridge, MA, USA). Polyclonal antibodies to matrix metalloprotease (MMP)9 were from Biomol (Hamburg, Germany). Additional chemicals were from Sigma-Aldrich Chemical Corporation (Taufkirchen, Germany).

### General methods

The hyaluronan synthase activity was determined by incorporation of radioactive [^14^C]glucuronic acid from UDP- [^14^C]GlcA and UDP-GlcNac [7]. The cytotoxicity of the drugs was measured as described previously [34]. For all experiments, the weight of the explants was determined immediately after cutting to minimize evaporation and the data were related to wet weight.

### Determination of hyaluronan

Cartilage explants were incubated in the absence or presence of IL-1 (2 ng/ml) and the inhibitors at various concentrations in serum-free Dulbecco's medium for 3 days. The amount of hyaluronan released into the culture medium was determined using biotinylated HABP, as described previously [30].

### Determination of proteoglycans

Cartilage explants were weighed (average wet weight 20 mg) and incubated in the absence and presence of IL-1 (2 ng/ml) and the inhibitors at various concentrations for 5 days. The tissues were extracted with 1.5 ml of a solution of 4 mol/l guanidinium hydrochloride, 0.1 mol/l ε-aminohexanoid acid, 5 mmol/l benzamidine, 10 mmol/l N-ethylmaleinimide and 0.5 mmol/l phenalmethylsulfonyl fluoride for 3 days at 4°C. The solution was centrifuged for 5 minutes at 10.000 *g *and the proteoglycans were determined in the supernatant using the alcian blue method, as described previously [35].

### Determination of the proteoglycan synthesis

Chondrocytes were cultured in alginate beads, as described above, and supplemented with 25 μl [^35^S]sulphate (0.5 mCi/ml) for 24 hours. The beads were washed three times with 102 mmol/l CaCl_2 _to remove un-incorporated radioactivity and dissolved in 55 mmol/l sodium citrate. Proteoglycans were isolated by the alcian blue precipitation method [36] and aliquots were used in the determination of radioactivity.

### Measurement of degraded collagen

The procedure for measurement of degraded collagen is described in detail in the report by Kosaki and coworkers [37]. Cartilage explants were cultured in Dulbecco's medium for 28 days in the presence or absence of IL-1 (2 ng/ml), IL-17 (25 ng/ml), 2 μmol retinoic acid and hyaluronan export inhibitors, and media were changed every 2 days. The cartilage was weighed and extracted with 4 mol/l guanidinium hydrochloride in 0.1 mol/l Tris HCl (pH 7.3), 1 mmol/l Iodoacetamide, 1 mmol/l EDTA, and 10 μg/ml pepstatin A for 72 hours. The extracted explants were washed with 1 mmol/l iodoacetamide and 1 mmol/l EDTA, and 10 μg/ml pepstatin in 0.1 mol/l Tris-HCl (pH 7.3) three times for 2 hours. The denatured collagen was digested overnight at 37°C with a solution of 0.5 ml of α-chymotrypsin (0.5 mg/ml) in 1 mmol/l iodoacetamide and 1 mmol/l EDTA, and 10 μg/ml pepstatin in 0.1 mol/l Tris-HCl (pH 7.3). The mixture was centrifuged for 8 minutes at 10,000 *g*, and the supernatant containing the digested collagen was separated from the remaining insoluble matrix containing the intact collagen. The insoluble material was hydrolyzed with 500 μl of 6 mol/l HCl at 110°C for 20 hours. The hydrolysate was neutralized with 500 μl of 6 mol/l NaOH and undissolved material was removed by centrifugation. The amount of the collagen-specific amino acid hydroxyproline was determined. An aliquot (25 μl) was mixed with 975 μl citrate buffer (57 g sodium acetate, 37.5 g sodium citrate, 5.5 g citric acid and 385 ml 2-propanol in 1 l water). An aliquot (200 μl) of this mixture was added to 100 μl of a solution of 100 mg chloramine T in 1 ml water, 2 ml 2-propanol and 3 ml citrate buffer. After 20 minutes at room temperature, 100 μl of 6.2 mol/l perchloric acid was added and reacted for 12 minutes at room temperature. A solution of 100 μl Ehrlichs reagent (500 mg in 1.25 ml ethanol and 1.25 ml diethyleneglycol-monoethylether) was added and incubated at 60°C for 20 minutes. The adsorption was read at 565 nm and the content of hydroxyproline was calculated using 1 to 30 μg/ml calibration samples.

### Determination of collagen synthesis

Chondrocytes were cultured in alginate beads for 1 week with 10% foetal calf serum in Dulbecco's medium. The medium was changed and supplemented with 1 mmol/l cysteine, 1 mmol/l pyruvate, 60 μg/ml β-aminopropionitril and 25 μg/ml ascorbic acid, and the beads were incubated for an additional 24 hours. The medium was replaced with serum-free medium containing the above supplements, IL-1, the inhibitors and [^14^C]proline (2 μCi/ml), and the cells were incubated for 24 hours. The beads were washed three times with 0.9% NaCl and CaCl_2 _(116 mg/l) for 30 minutes to remove unincorporated radioactivity, dissolved in 500 μl of 55 mmol/l sodium citrate, and the radioactivity was determined.

### Zymography of matrix proteases

Bovine cartilage explants were cultured in serum-free Dulbecco's medium for 5 days in the presence or absence of IL-1α (2 ng/ml) and 10 or 30 μmol/l zaprinast, vardenafil, or tadalafil. The protein concentrations of the culture media were determined and equal amounts of proteins were directly applied to a 7.5% SDS-polyacrylamide gel that contained 0.1% gelatin. After electrophoresis, the gel was washed twice with 2.5% Triton X-100 for 30 minutes, three times with water for 10 minutes, and incubated in a solution of 50 mmol/l Tris-HCl, 5 mmol/l CaCl, 1 μmol/l ZnSO_4 _(pH 8.0) for 5 days at 37°C. The gel was stained with Coomassie blue.

### Determination of protein infiltration into the cartilage matrix

Bovine cartilage explants were cultured in Dulbecco's medium and 10% foetal calf serum in the presence or absence of IL-1α (2 ng/ml) and the drugs zaprinast, tadalafil and vardenafil for 14 days. Pieces of 2 mm diameter and a volume of 3.15 mm^3 ^were punched out. They were incubated in a solution of 10 units/ml of horseradish peroxidase (HRPO) in phosphate-buffered saline for 1 hour at 37°C. The pieces were rinsed with water and shaken in 250 μl phosphate-buffered saline overnight at 4°C to release the infiltrated enzyme. The concentrations of HRPO were determined in 50 μl of the supernatants. A solution (150 μl) of ABTS (2,2'-azino-bis [3-ethylbenzthiazoline-6-sulfonic acid]; 1 mg/ml) and 0.03% H_2_O_2 _was added, and after incubation for 30 minutes at 37°C the adsorbance at 405 nm was read.

### Statistical analyses

Data are presented in the figures as mean ± standard deviation. The *t*-test was used, and a *P *value below 0.05 was considered statistically significant.

## Results

### Inhibition of hyaluronan export

**Figure 1:**
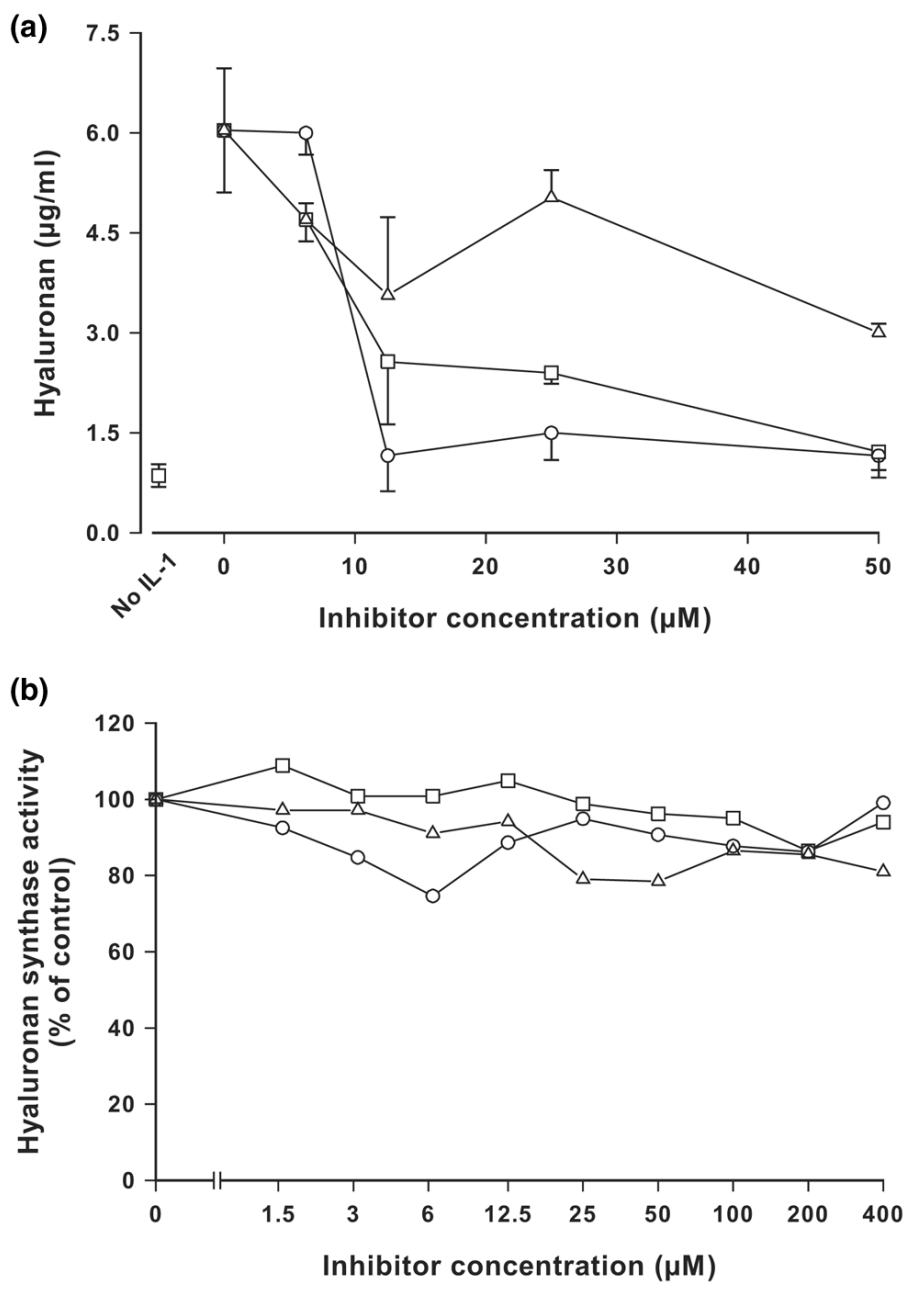
Inhibition of hyaluronan export in bovine chondrocytes. **(a) **Cartilage explants were incubated in the absence and presence of IL-1α and the drugs tadalafil (○), zaprinast (□), or vardenafil (△). The concentration of hyaluronan was determined in the supernatant after 3 days. The error bars indicate the standard deviation of three determinations. **(b) **Effect of inhibitors on hyaluronan synthase activity. A particulate fraction of chondrocytes was prepared and incubated with the substrates UDP-GlcNac and UDP-[^14^C]GlcA and increasing concentrations of tadalafil (○), zaprinast (□), or vardenafil (△), and the incorporation into [^14^C]hyaluronan was determined.

The drugs tadalafil, zaprinast and vardenafil were analyzed for their effects on hyaluronan export from bovine cartilage explants in tissue culture. Cartilage explants were incubated for 3 days in the presence and absence of IL-1α and increasing concentrations of the drugs. Figure 1a shows that IL-1α stimulated an increase in hyaluronan export by about sixfold, and the inhibitors partially reversed it. In control experiments, the inhibitors were analyzed for their effect on the hyaluronan synthase activity of chondrocytes cultured in alginate beads. Activity was reduced by less than 20% up to concentrations of 400 μmol/l (Figure 1b). The toxicity of the drugs was less than 10% at a concentration of 100 μmol/l for the three inhibitors.

### Inhibition of proteoglycan loss

**Figure 2:**
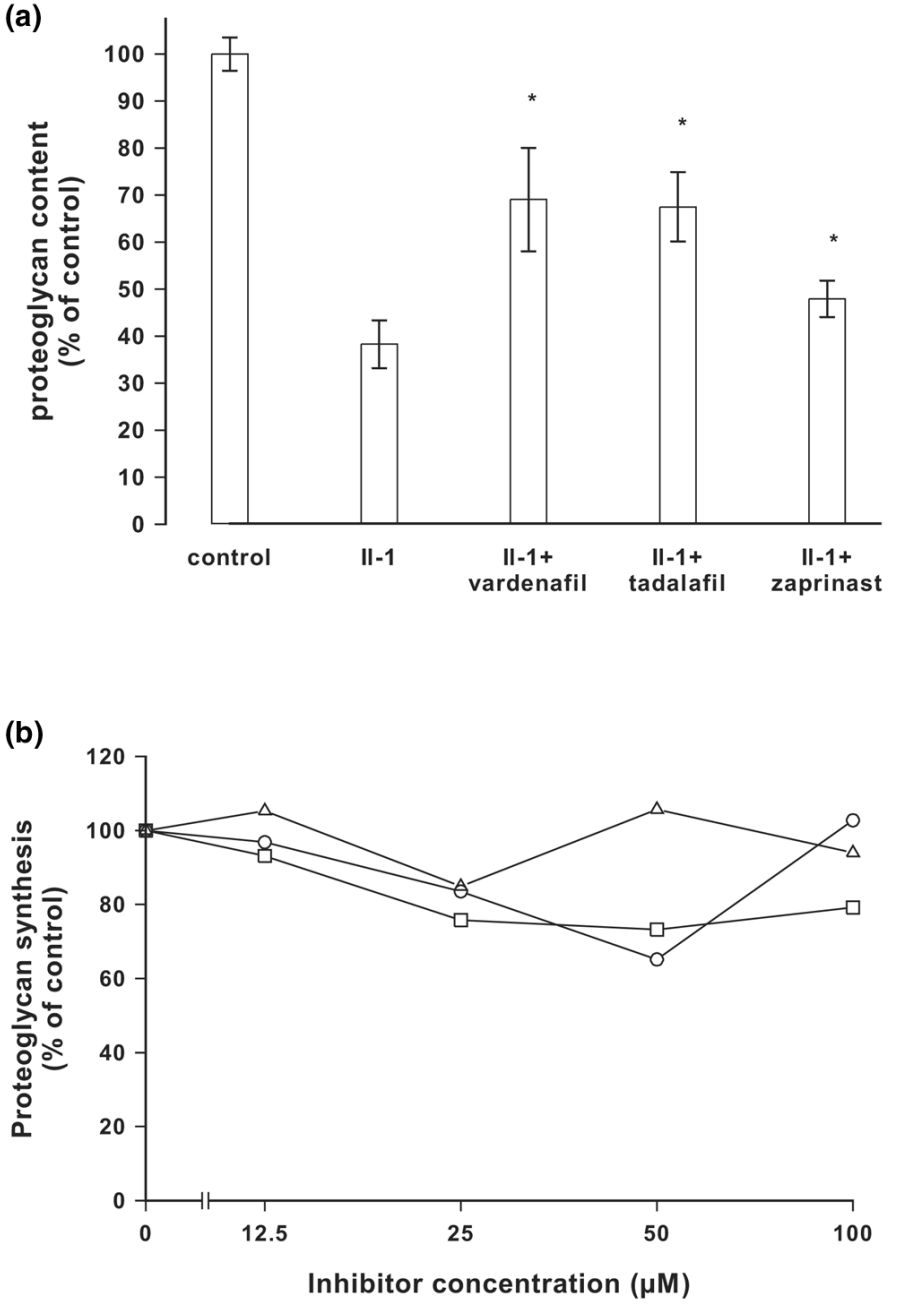
Inhibition of proteoglycan loss in bovine chondrocytes. **(a) **Cartilage explants were incubated in the absence and presence of IL-1α and the drugs tadalafil, zaprinast, or vardenafil at concentrations of 50 μmol/l. The tissues were weighed, extracted with guanidinium hydrochloride, and the amount of proteoglycans was determined after 5 days. The data were related to controls without IL-1α as 100%. The error bars represent the standard deviation of three determinations; *P < 0.05. **(b) **Effect of inhibitor tadalafil (○), zaprinast (□), or vardenafil (△) on proteoglycan synthesis. Bovine chondrocytes were cultured in alginate beads and incubated with increasing concentrations of the inhibitors in the presence of [^35^S]sulphate. After 24 hours the radioactivity incorporated into [^35^S]proteoglycans was determined.

The drugs tadalafil, zaprinast and vardenafil were analyzed for their effects on proteoglycan loss from IL-1α activated bovine cartilage explants. Proteoglycans were extracted from the tissues with guanidinium hydrochloride and determined colourimetrically. Figure 2a shows that IL-1α reduced the proteoglycan content in cartilage to less than 40% of that in the untreated control. The inhibitors protected the cartilage from proteoglycan loss. In a control experiment, the effect of the inhibitors on the proteoglycan synthesis rate was determined. Bovine chondrocytes were cultured in alginate beads and incubated with [^35^S]sulphate in the presence of drugs, and inhibition of proteoglycan synthesis was found to be reduced by less than 25% (Figure 2b). These findings confirm earlier observations obtained with other drugs [30] and suggest that zaprinast, vardenafil and tadalafil prevented proteoglycan loss from osteoarthritic cartilage primarily by inhibition of hyaluronan over-production.

### Inhibition of collagen degradation

**Figure 3:**
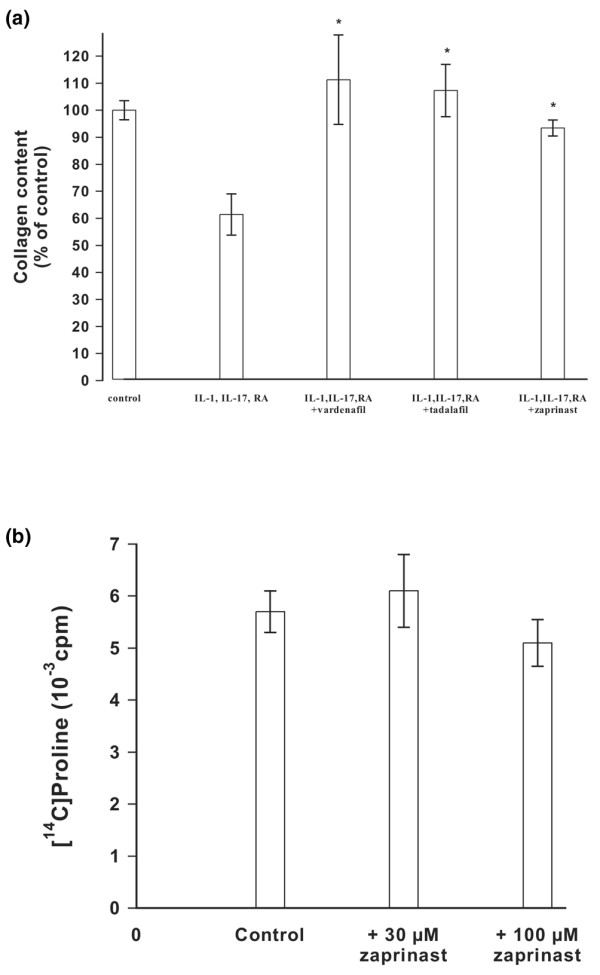
Quantitative analysis of collagen degradation and its inhibition. **(a) **Cartilage explants were incubated with IL-1α, IL-17 and retinoic acid in the absence or presence of zaprinast, tadalafil, or vardenafil at concentrations of 50 μmol/l for 4 weeks at 37°C. The amount of chymotrypsin-resistant collagen was determined as hydroxyproline. The values were related to the control that contained 23 μg hydroxyproline/mg cartilage as 100%. The bars indicate the standard deviation of four determinations; *P < 0.05. **(b) **Bovine chondrocytes were grown in alginate beads and and collagen was labeled by incorporation of [^14^C]proline in the absence or presence of 30 μmol/l and 100 μmol/l zaprinast. The amount of [^14^C]collagen within the alginate beads was determined after 24 hours. The bars indicate the standard deviation of four determinations.

The drugs were analyzed for their effects on collagen degradation in IL-1α activated cartilage explants. Preliminary experiments revealed that induction of osteoarthritic reactions by IL-1α was not sufficient to detect measurable amounts of collagen degradation products. Degradation can be enhanced substantially by addition of IL-17 and retinoic acid. Therefore, these activators were added. Activated cartilage explants were incubated with tadalafil, zaprinast, or vardenafil for 28 days, extracted with guanidinium hydrochloride, and digested with chymotrypsin. Degraded collagen was measured as the amount of hydroxyproline that was susceptible to chymotrypsin. Figure 3a shows that cartilage activation reduced the amount of chymotrypsin-resistant collagen to 65%. Inhibition of hyaluronan export restored the content of intact collagen. In a control experiment, the effect of zaprinast on collagen synthesis was measured (Figure 3b). Bovine chondrocytes were cultured in alginate beads and incubated in culture medium containing [^14^C]proline in the absence and presence of zaprinast and incorporation of radioactivity into pepsin-resistant collagen was determined. The total amount of collagen was not altered significantly at concentrations up to 100 μmol/l zaprinast. These findings suggest that the drugs did not affect collagen synthesis and that the protection from collagen degradation could involve other mechanisms.

### Inhibition of the action of gelatinases

**Figure 4:**
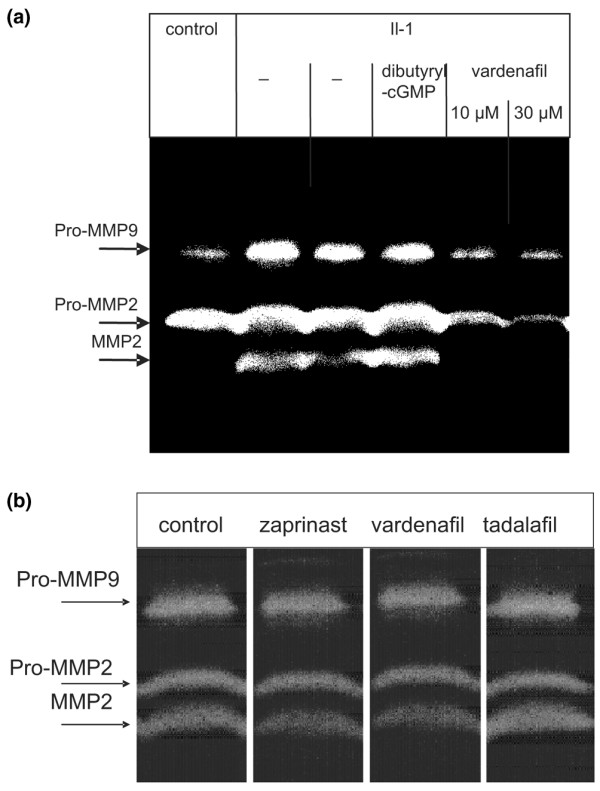
Effect on gelatinases. **(a) **Inhibition of gelatinase liberation from bovine cartilage explants. Cartilage explants were incubated in the absence or presence of IL-1α and 25 μmol/l dibutyryl-cGMP or 10 μmol/l or 30 μmol/l vardenafil for 4 days at 37°C. The lanes marked with (-) indicate two independent control experiments with IL-1 only. The activity of gelatin-degrading enzymes released into the culture supernatant was determined by zymography. **(b) **Unaffected gelatinase synthesis. Unstimulated explants were incubated for 10 days in the absence and presence of 100 μmol/l zaprinast, vardenafil and tadalafil, and the gelatinase activities were again analyzed by gel zymography. MMP, matrix metalloprotease.

A possible explanation for the protective effect of hyaluronan export inhibition on collagen degradation could be that the altered composition and permeability of osteoarthritic cartilage allowed the diffusion of metalloproteases. It is known that chondrocytes produce gelatinases, particularly if they are activated by IL-1 [38]. We tested this possibility by measuring the release of gelatinases from IL-1α activated cartilage. Cartilage explants were incubated in the absence and presence of IL-1α and vardenafil, and enzymes released from the cartilage explants were analyzed by gel zymography. We also included dibutyryl-cGMP in the analysis, because cGMP has been shown to mediate IL-1 signalling in chondrocytes [39]. Figure 4a shows three bands with molecular weights of 86 kDa, 66 kDa and 62 kDa. The upper band was probably pro-MMP9, because it reacted with monoclonal antibodies in Western blots (data not shown). The lower two bands comigrated with an authentic sample of pro-MMP2 and MMP2 gelatinases (from Dr R Dreier; data not shown). IL-1α enhanced the release of the gelatinases, and this release was not significantly altered by addition of dibutyryl-cGMP. Vardenafil reduced the gelatinase release in a concentration-dependent manner. Similar results were obtained with zaprinast and tadalafil (data not shown).

In a control experiment, we analyzed whether inhibition of hyaluronan export altered the syntheses of gelatinases by chondrocytes in unstimulated cartilage explants. The explants were incubated in the absence and presence of 100 μmol/l zaprinast, vardenafil and tadalafil, respectively, and the gelatinase activities were again analyzed by gel zymography. Figure 4b shows no differences in enzyme activities between the samples. These results showed that inhibition of hyaluronan export in IL-1 activated cartilage explants inhibited the release of gelatinases into the culture medium.

### Inhibition of protein diffusion through cartilage explants

**Figure 5:**
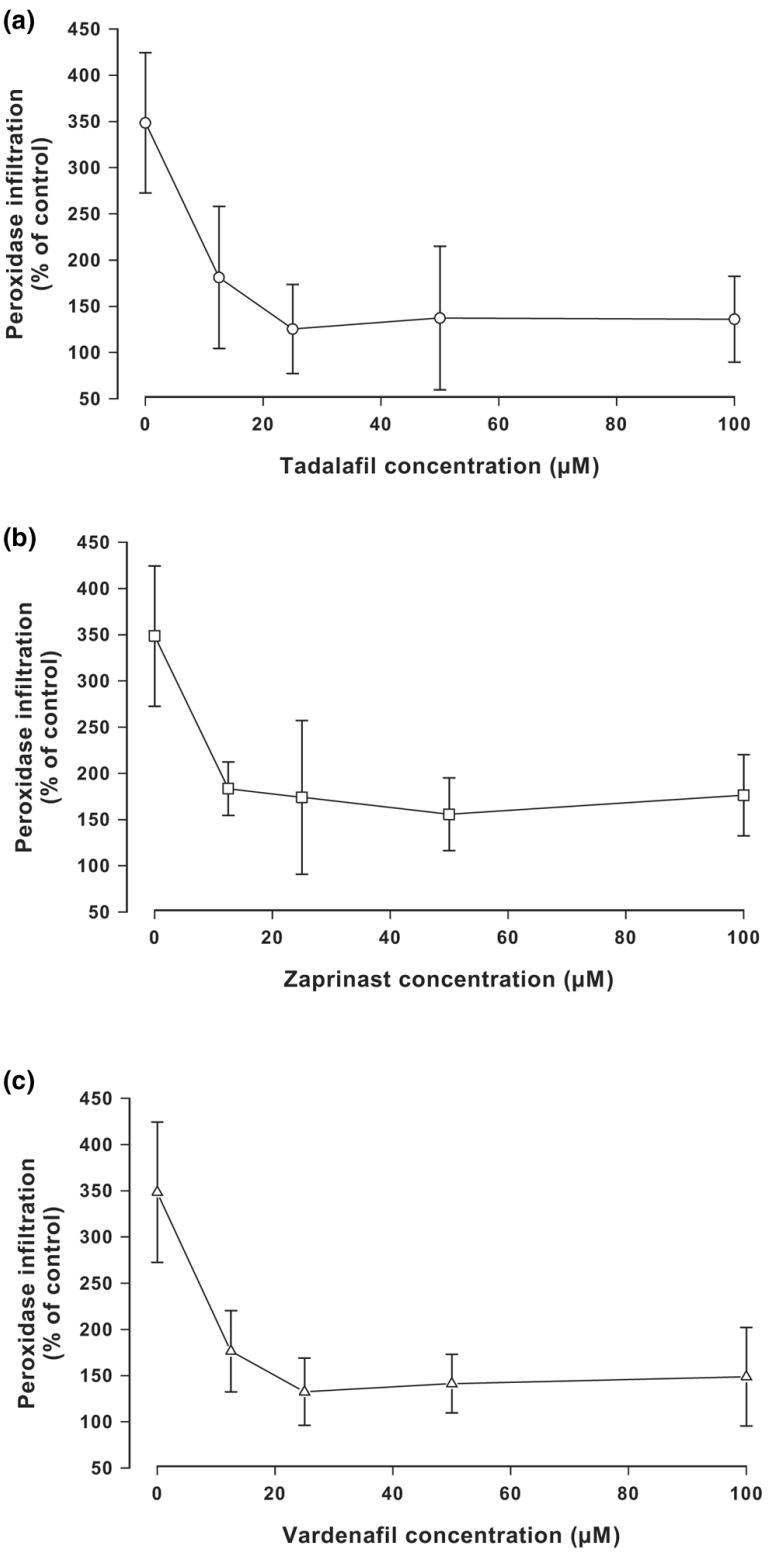
Inhibition of protein infiltration into bovine cartilage explants. Cartilage explants were incubated in the absence or presence of IL-1α and **(a) **tadalafil, **(b) **zaprinast, or **(c) **vardenafil for 14 days at 37°C. The explants were then incubated with horseradish peroxidase as indicator protein for 1 hour. After washing, the amount of enzyme that had infiltrated the explants was determined by a colour reaction. The error bars represent the standard deviation of eight determinations.

If the altered composition of arthritic cartilage with increased hyaluronan and decreased proteoglycan content was responsible for facilitated diffusion of degrading enzyme through the matrix, exogenous enzymes should infiltrate better. This hypothesis was tested using HRPO as an indicator protein. Cartilage explants were incubated in the absence or presence of IL-1α and zaprinast, vardenafil, or tadalafil in increasing concentrations. The explants were incubated with the indicator protein HRPO to allow diffusion into the cartilage. After extensive washing, the explants were further incubated in phosphate-buffered saline to liberate the infiltrated enzyme. The amount of liberated enzyme was determined by a colour reaction. Control experiments indicated that the drugs did not have any direct effect on the peroxidase activity at micromolar concentrations. Figure 5 shows that IL-1α treatment led to an increase of enzyme infiltration of about 350% over the unstimulated control (100%). Inhibition of hyaluronan export reduced the IL-1α induced enzyme infiltration almost to control values.

### Mechanism of inhibitor action

**Figure 6:**
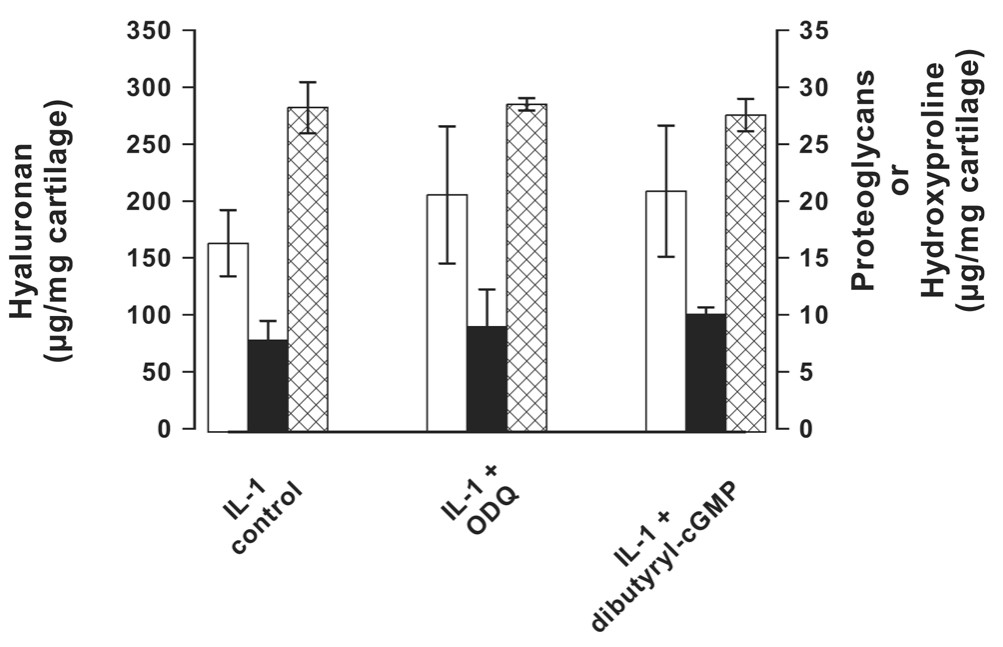
Influence of cGMP modulators. Shown is the influence of cGMP modulators on the hyaluronan (open bars), proteoglycan (solid bars), and collagen (cross-hatched bars) production of bovine cartilage. Cartilage explants were cultured in the presence of 2 ng/ml IL-1 and the guanylate cyclase inhibitor ODQ (1H-[1,2,4]-oxadiazolo [4,3a]quinoxaline-1-one; 25 μmol/l) or dibutyryl-cGMP (25 μmol/l). The incubation periods were for 3 days for hyaluronan, 5 days for proteoglycans and 28 days for collagens. For stimulation of collagen degradation, the cartilage explants were supplemented with 25 ng/ml IL-17, and 2 μmol/l retinoic acid. The concentrations of hyaluronan in the supernatant, proteoglycans and collagen (as hydroxyproline) were determined as described under Materials and methods. The error bars represent the standard deviation of three determinations.

There are two possible mechanisms for the inhibitory action of the drugs zaprinast, vardenafil and tadalafil. Because of their PDE5 inhibitory activity, with Ki values of 300 nmol/l for zaprinast, 1.5 nmol/l for vardenafil and 2.9 nmol/l for tadalafil [40,41], they will certainly raise the concentration of intracellular cGMP that could inhibit hyaluronan export by MRP5 [8]. It is also possible that they additionally act as MRP5 inhibitors, because they are structural analogues of cGMP; also, it is known that zaprinast inhibits transport at concentrations between 20 nmol/l and 250 μmol/l, depending on the transported substrate [33,42,43]. If the drugs acted only through inhibition of PDE5, then also other unrelated PDE5 inhibitors or addition of other cGMP analogs should have similar effects. We therefore analyzed hyaluronan export, proteoglycan loss and collagen degradation of IL-1 activated cartilage explants in the presence of dibutyryl-cGMP and ODQ (1H-[1,2,4]-oxadiazolo [4,3a]quinoxaline-1-one), which is a selective inhibitor of the soluble nitric oxide inducible guanylate cyclase. Figure 6 shows that none of the parameters was significantly altered by these treatments. Similar results were obtained with bromo-cGMP (data not shown). These findings suggest that alterations of the intracellular cGMP concentration did not account for the inhibitory effects of the drugs zaprinast, vardenafil and tadalafil.

## Discussion

One of the earliest events in the pathogenesis of osteoarthritis is hyaluronan over-production of chondrocytes that precedes the stimulation of protease synthesis, collagen degradation and cartilage destruction [44-46]. It can be induced by IL-1 treatment in cell and organ culture and in animal models of osteoarthritis. IL-1 alters the cartilage composition by influencing the transcription rate of enzymes and matrix components [47].

In a previous report we showed that hyaluronan over-production led to loss of proteoglycans from osteoarthritic cartilage [30]. Inhibition of hyaluronan over-production normalized the proteoglycan content on alginate cultures of bovine chondrocytes, in cartilage explants and in an animal model of osteoarthrosis. We showed that enhanced intracellular cGMP levels reduce hyaluronan export from fibroblasts [8]. In the present study we extended the effect of hyaluronan export inhibitors to collagen degradation. We used the drugs zaprinast, vardenafil and tadalafil that were originally developed as PDE5 inhibitors [32].

We showed here that the drugs inhibited hyaluronan export, and protected cartilage from proteoglycan loss, release of metalloproteases into the medium and collagen degradation. The drugs did not influence substantially the rates of synthesis of hyaluronan, proteoglycans, metalloproteases and collagens. Our experiments also suggest a mechanism for how the inhibitors of hyaluronan export were able to prevent collagen degradation. Hyaluronan over-production increased the infiltration of the indicator protein HRPO into IL-1α treated cartilage, and this infiltration was reduced by inhibition of hyaluronan export. It is likely that this inhibition also applies to reduced diffusion of proteoglycans and gelatinases MMP2 and MMP9 or other degrading enzymes out of the cartilage or from their origin to the targets within the cartilage.

The reason for the enhanced diffusion of proteins through osteoarthritic cartilage can be found in the altered cartilage composition. Proteoglycans at high concentrations in cartilage play a critical role in the flow and diffusion of macromolecules. Because of the high density of fixed charges, they vigorously restrict diffusion [48,49]. If the dense packing of proteoglycans is lost and replaced by voluminous hyaluronan, degrading enzymes can freely reach their targets. It has indeed been demonstrated that aggrecan protects cartilage collagen from proteolytic degradation [50]. As a consequence of this scenario, collagen is protected from degradation by inhibition of hyaluronan export.

We also analyzed the mechanism of hyaluronan export inhibition. At nanomolar concentrations, the PDE5 inhibitors substantially elevate the intracellular cGMP levels [32]. Zaprinast acts also as a MRP5 inhibitor in micromolar concentrations [33]. Because the effects that we observed on hyaluronan export, proteoglycan loss and collagen degradation were all found in the micromolar range, it is likely that the drugs primarily exerted their effects on export by MRP5 rather than through an increase in intracellular cGMP levels. Although based on a limited dataset, the rank order of potency for PDE5 inhibition of zaprinast (Ki = 300 nmol/l), tadalafil (Ki = 2.9 nmol/l) and vardenafil (Ki = 1.5 nmol/l) does not correlate with the apparent potency of these compounds in inhibiting hyaluronan export induced by IL-1 (Figure 1). This notion was supported by our experiments that altered the intracellular cGMP by the specific guanylate cyclase inhibitor ODQ and the analogues dibutyryl-cGMP and bromo-cGMP. These compounds did not have any significant effect on hyaluronan export, proteoglycan loss and collagen degradation. The lack of an effect by cGMP analogues on chondrocytes is surprising and clearly different from the effects on fibroblasts [8]. Such cell-dependent discrepancies of MRP5 inhibition were previously observed in other cell lines and are probably due to different MRP5 copy numbers per cell [43].

The drugs zaprinast, tadalafil and vardenafil have been developed for other disturbances and are certainly not ideal for treatment of osteoarthrosis. It may be worthwhile to develop specific hyaluronan export inhibitors, because they could not only prevent proteoglycan loss and collagen degradation, but also inhibit subsequent reactions that lead to apoptosis of chondrocytes.

## Conclusion

Inhibition of hyaluronan export from chondrocytes attenuated proteoglycan loss, collagen degradation, protein diffusion and metalloprotease activity in IL-1 activated cartilage and could be effective in osteoarthrosis.

## Abbreviations

HABP = hyaluronan binding protein; HRPO = horseradish peroxidase; IL = interleukin; MMP = matrix metalloprotease; MRP = multidrug resistance-associated protein; ODQ = 1H-(1,2,4)-oxadiazolo (4,3a)quinoxaline-1-one.

## Competing interests

The authors declare that they have no competing interests.

## Authors' contributions

BD performed the experiments, evaluated the data and designed the experiments. PP evaluated the data, designed experiments and wrote the manuscript.
